# Complete genome sequencing and phylogenetic analysis of dengue type 1 virus isolated from Jeddah, Saudi Arabia

**DOI:** 10.1186/s12985-014-0235-7

**Published:** 2015-01-16

**Authors:** Esam I Azhar, Anwar M Hashem, Sherif A El-Kafrawy, Said Abol-Ela, Adly MM Abd-Alla, Sayed Sartaj Sohrab, Suha A Farraj, Norah A Othman, Huda G Ben-Helaby, Ahmed Ashshi, Tariq A Madani, Ghazi Jamjoom

**Affiliations:** Special Infectious Agent Unit, King Fahd Medical Research Center, King Abdulaziz University, Jeddah, Kingdom of Saudi Arabia; Department of Medical Laboratory Technology, Faculty of Applied Medical Sciences, King Abdulaziz University, Jeddah, Kingdom of Saudi Arabia; Department of Medical Microbiology and Parasitology, Faculty of Medicine, King Abdulaziz University, Jeddah, Kingdom of Saudi Arabia; Insect Pest Control Laboratory, Joint FAO/IAEA Program of Nuclear Techniques in Food and Agriculture, Vienna, Austria, Pests and Plant Protection Department, National Research Center, Cairo, Egypt; Department of Laboratory Medicine, Faculty of Applied Medical Science, Umm Al-Qura University, Makkah, Kingdom of Saudi Arabia; Department of Medicine, Faculty of Medicine, King Abdulaziz University, Jeddah, Kingdom of Saudi Arabia

**Keywords:** Dengue virus, Full genome, Phylogenetic analysis, Diversity, Jeddah, Saudi Arabia

## Abstract

**Background:**

Dengue viruses (DENVs) are mosquito-borne viruses which can cause disease ranging from mild fever to severe dengue infection. These viruses are endemic in several tropical and subtropical regions. Multiple outbreaks of DENV serotypes 1, 2 and 3 (DENV-1, DENV-2 and DENV-3) have been reported from the western region in Saudi Arabia since 1994. Strains from at least two genotypes of DENV-1 (Asia and America/Africa genotypes) have been circulating in western Saudi Arabia until 2006. However, all previous studies reported from Saudi Arabia were based on partial sequencing data of the envelope (E) gene without any reports of full genome sequences for any DENV serotypes circulating in Saudi Arabia.

**Findings:**

Here, we report the isolation and the first complete genome sequence of a DENV-1 strain (DENV-1-Jeddah-1-2011) isolated from a patient from Jeddah, Saudi Arabia in 2011. Whole genome sequence alignment and phylogenetic analysis showed high similarity between DENV-1-Jeddah-1-2011 strain and D1/H/IMTSSA/98/606 isolate (Asian genotype) reported from Djibouti in 1998. Further analysis of the full envelope gene revealed a close relationship between DENV-1-Jeddah-1-2011 strain and isolates reported between 2004–2006 from Jeddah as well as recent isolates from Somalia, suggesting the widespread of the Asian genotype in this region.

**Conclusions:**

These data suggest that strains belonging to the Asian genotype might have been introduced into Saudi Arabia long before 2004 most probably by African pilgrims and continued to circulate in western Saudi Arabia at least until 2011. Most importantly, these results indicate that pilgrims from dengue endemic regions can play an important role in the spread of new DENVs in Saudi Arabia and the rest of the world. Therefore, availability of complete genome sequences would serve as a reference for future epidemiological studies of DENV-1 viruses.

## Background

Dengue virus (DENV) is a positive sense single-stranded RNA virus belonging to the *Flaviviridae* family. It is an endemic mosquito-borne virus affecting more than 50 million people globally [1]. Infection with any DENV serotype (DENV 1–4) can range from asymptomatic infection to mild dengue fever. On the other hand, re-infection with heterologous serotypes can lead to more severe disease, i.e. dengue with warning signs or severe dengue infection [2,3].

DENV genome varies in size from 10.6 to 11 kb and encodes three structural and seven non-structural proteins [4]. The structural proteins are comprised of the capsid (C), membrane (M) and envelope (E) proteins, and the non-structural proteins include the NS1, NS2A, NS2B, NS3, NS4A, NS4B and NS5. DENV genome is flanked by 94 nucleotides (nts) at 5′ untranslated regions (UTR) and 388–462 nts at the 3′ UTR [5]. While the four DENV serotypes share 65–70% sequence homology, they are further clustered into different genotypes due to the high mutation rates [6,7].

Epidemiological and phylogenetic studies have shown geographical movement and divergence of DENVs [8-10]. Such studies have also shown an association between certain genotypes and disease severity. Shifts in circulating genotype or introduction of new genotypes or virulent strains in endemic regions have been shown to be linked with increased severity and occurrence of severe dengue infection [11,12]. Therefore, it is critically important to monitor circulating DENVs in endemic countries to better understand the epidemiology of the disease and to design and implement disease surveillance programs.

Millions of Muslims, from all over the world, visit Makkah and Al-Madinah every year to perform Hajj and Umrah. Jeddah city is the connection terminal where pilgrims gather on their way to the holy places in Makkah and Al-Madinah. Thus, pilgrims arriving from dengue endemic countries can play a major role in the introduction of new DENVs into Saudi Arabia. The large number of expatriates particularly from the Indian subcontinent, Southeast Asia and Africa, who work in Saudi Arabia and represent ~20% of the population, as well as the leisure travel to dengue endemic countries in Southeast Asia, which is very common among Saudi citizens, represent other major sources of DENV importation into Saudi Arabia. All these factors could put Saudi Arabia at the crossroads for global spread of DENVs.

Several outbreaks have been reported in Saudi Arabia and neighboring country Yemen [13-20]. In 1994, DENV-1 and DENV-2 were reported to cause major outbreaks which were followed by the emergence of DENV-3 in 1997 [19]. Since then, all 3 serotypes (DENV 1–3) were being reported and isolated in Saudi Arabia [13-20]. Phylogenetic analysis indicate that recent isolates from both DENV-2 and DENV-3 belong to cosmopolitan genotype and genotype III, respectively, and there has been no major genetic changes since their first detection in Saudi Arabia [20]. On the other hand, most recent DENV-1 isolates belong to Asia genotype which might have replaced the pre-circulating America/Africa genotype that was first detected in 1994, although their co-circulation cannot be excluded [20]. Nonetheless, previous studies from Saudi Arabia have mainly focused on the analysis of small genomic sequences in the E⁄NS1 junction or the E gene of DENVs for the determination of genetic variation and molecular characterization of isolated strains [20]. Furthermore, while several complete genome sequences of DENV-1 were reported for many strains around the world, no complete sequence for any DENV-1 isolated from Saudi Arabia has been reported yet. Here, we report the first complete genome and detailed genetic analysis of DENV-1 isolate obtained from a patient in Jeddah in Saudi Arabia in 2011 which should provide reference data for future genetic studies on DENV-1 in Saudi Arabia and the region.

## Results

### Laboratory diagnosis

**Figure 1:**
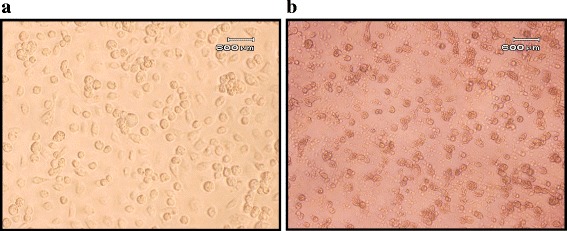
**Dengue infected C6/36 cells. a**. Uninfected C6/36 cells. **b**. CPE 5 days after inoculation of C6/36 cells with patient plasma.

A 26-year-old previously healthy Saudi women presented to the Emergency Department of King Abdulaziz University Hospital with primary dengue-like symptoms including fever, headache, myalgia, joint pain, nausea and vomiting. Laboratory investigations of the patient revealed a low platelet count of 63 × 10^3^ cells/mm^3^ (150–450 × 10^3^ cells/mm^3^) and elevated liver enzymes levels; alanine amino transferase (ALT) 264 U/L (7–55 U/L), aspartate amino transferase (AST) 326 U/L (8–48 U/L) and gamma glutaryl transferase (GGT) 240 U/L (9–48 U/L). Further analysis showed that the patient’s serum was positive for anti-dengue IgM but not anti-dengue IgG antibodies indicating an acute primary dengue infection. Real time RT-PCR confirmed these results and viral RNA was detected in the patient's plasma. Upon inoculation of plasma in C6/36 cells, cytopathic effect (CPE) was observed after 5 days (Figure 1) and the cell culture supernatant tested positive for DENV RNA by real time RT-PCR.

### Complete genome sequence and phylogenetic analysis

In order to characterize the DENV-1-Jeddah-1-2011 isolate, the full genome was sequenced. Assembly of all sequenced overlapping fragments obtained by RT-PCR resulted in a genome of 10,622 nts with a complete coding region encoding for 3392 amino acids (aa) flanked by 94 and 349 nts at the 5’ UTR and 3’ UTR, respectively. Identity matrix of nts and aa of DENV-1-Jeddah-1-2011 isolate was then compared to highly similar isolates (38 DENV-1 strains) available in GenBank. As shown in Table 1, sequence identity matrix of DENV-1-Jeddah-1-2011 isolate genome ranged from 97.2-89.6% compared to other strains. On the other hand, aa identity was higher and ranged from 99.4-96.5% (Table 1). Interestingly, the highest identity was with AF298808-Djibouti-1998 strain (97.2% nt identity and 99.4% aa identity) followed by JN638336-Thailand-1986 strain (97.1% nt identity and 99.2% aa identity) and AB608788-Taiwan-1994 strain (96.7% nt identity and 99% aa identity).

**Table 1:** **Nucleotide (NT) and amino acid (AA) identity of DENV-1-Jeddah-1-2011 full genome compared to closely related strains**

**Strain**	**NT identity (%)**	**AA identity (%)**
AF298808-Djibouti-1998	97.2	99.4
JN638336-Thailand-1986	97.1	99.2
AB608788-Taiwan-1994	96.7	99.0
AF350498-China-2001	96.6	98.1
AY726555-Myanmar-1998	96.0	98.8
FJ469907-Singapore-2003	94.8	98.5
JN697058-Malaysia-2005	94.4	98.4
JN697057-Malaysia-2005	94.4	98.3
JN054255-SriLanka-2010	94.2	97.6
GQ398255-Singapore-2008	94.2	98.3
JN054256-SriLanka-2009	94.1	98.0
KF887994-Thailand-2013	94.1	98.0
AB074760-Japan-2001	93.9	97.5
KC759167-China-2012	93.8	97.7
JF459993-Myanmar-2002	93.5	98.2
KF955446-Vietnam-2008	92.9	96.5
AB074761-Japan-2001	91.5	97.6
AB189121-Indonesia-1998	91.2	97.3
AB189120-Indonesia-1998	91.2	97.5
KF184975-Angola-2013	91.1	97.0
FJ744701-Venezuela-2004	91.1	97.2
GQ868602-Phillipines-2004	91.1	97.4
JQ915076-French Polynesia:Tahiti-2009	90.8	97.3
JQ915074-French Polynesia:Moorea-2008	90.8	97.3
FJ850113-Nicaragua-2005	90.7	97.1
JX669475-Brazil-2002	90.6	97.0
JX669473-Brazil-2001	90.6	97.0
FJ810415-Venezuela-2005	90.6	97.1
JN903578-India-2007	90.6	97.0
FJ850114-Nicaragua-2005	90.4	97.0
KJ189368-Mexico-2012	90.2	96.9
JN903579-India-2008	90.2	96.8
KJ189369-Mexico-2011	90.0	97.1
KJ189366-Peurto Rico-2010	89.9	96.7
KJ189304-Colombia-2005	89.9	97.1
KJ189303-Colombia-1998	89.9	97.1
KJ189367-Peurto Rico-2010	89.8	96.9
KF921948-Vietnam-2008	89.6	97.6

Comparison of the genome size of DENV-1-Jeddah-1-2011 isolate (10,622 bp) with closely related isolates from other countries showed the AB608788-Taiwan-1994 strain as the closest in genome size (10693 bp) followed by AF298808-Djibouti-1998 (10721 bp) and JN638336-Thailand-1986 (10735 bp) strains. Comparing the coding region of DENV-1-Jeddah-1-2011 isolate to the closest strain, AF298808-Djibouti-1998, 159 nt mutations were found with 27 non-synonymous substitutions only. Most of the aa changes were clustered in the NS5 region of the viral protein with only 1-3 substitutions in other genes (Table 2).

**Table 2:** **Amino acid substitutions observed in the DENV-1-Jeddah-1-2011 isolate compared to AF298808-Djibouti-1998 isolate**

**S.N**	**Gene**	**Position**		**AA**	
		**AA**	**NT**	**DENV-1-Jeddah-1-2011**	**AF298808-Djibouti-1998**
1	**CP**	55	165	R	T
2	**PreM**	178	534	I	V
3	**E**	593	1779	V	A
4		618	1854	L	S
5	**NS1**	838	2514	L	V
6		855	2565	L	F
7		1129	3387	S	L
8	**NS2A**	1187	3561	V	I
9		1275	3825	A	E
10	**NS2B**	1378	4134	V	I
11	**NS3**	1536	4608	K	Q
12		1543	4629	T	S
13		2117	6351	S	N
14	**NS4A**	2170	6510	K	R
15		2187	6561	M	V
16	**NS5**	2350	7050	X	L
17		2364	7092	T	A
18		2628	7884	I	T
19		2758	8274	A	T
20		2941	8823	T	R
21		3013	9039	D	G
22		3035	9105	T	S
23		3120	9360	L	I
24		3133	9399	E	Q
25		3140	9420	G	E
26		3237	9711	G	E
27		3364	10092	K	R

**Figure 2:**
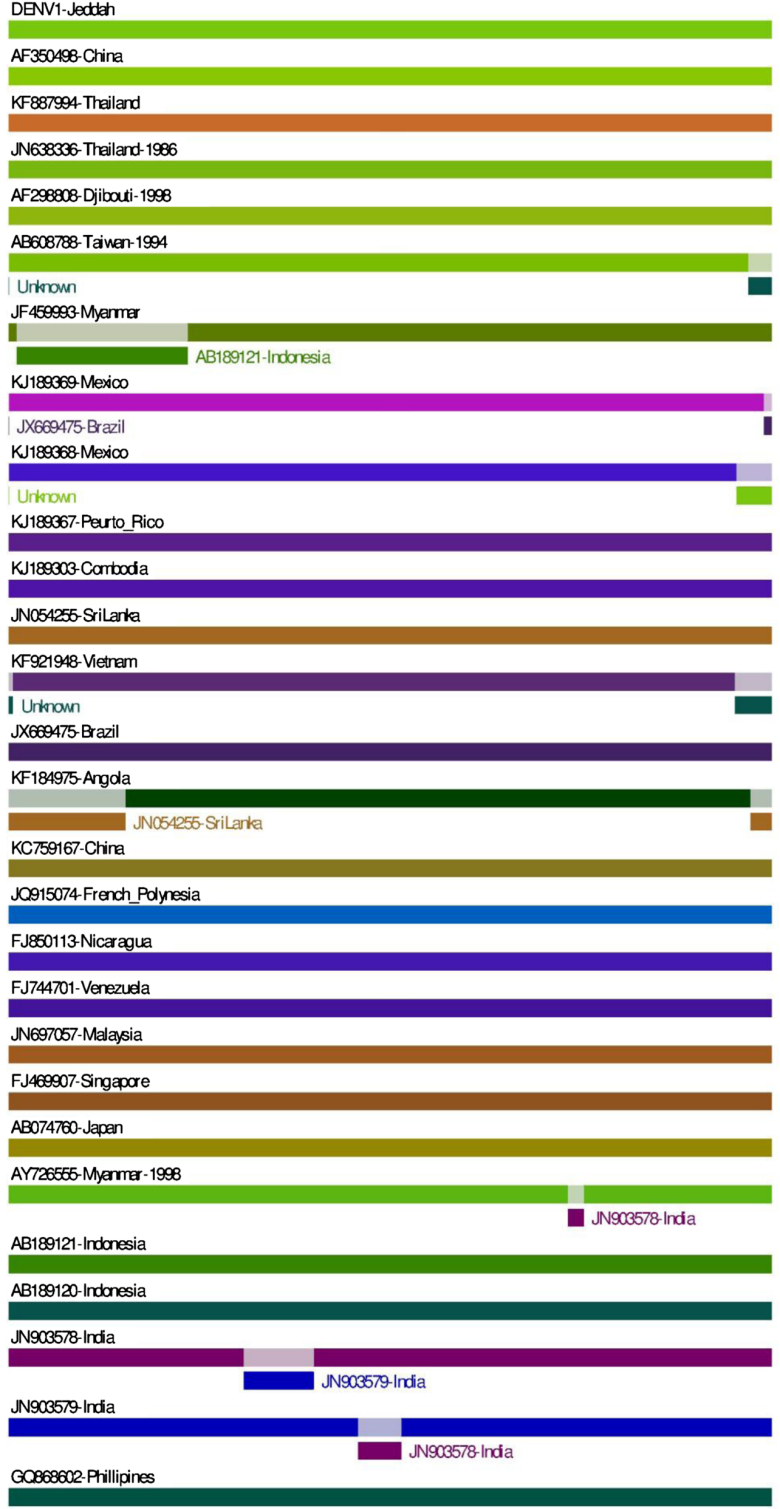
**Recombination events in DENV-1 strains.** Schematic representation indicates the recombination events in DENV-1 strains analyzed by RDP3 package with P-value < 0.05. Continues lines indicate no recombination possibility. Analysis was performed on all sequences included in the phylogenetic tree in Figure 3. The following strains did not show any recombination events and are not shown in the figure; KJ189366-Peurto Rico-2010, KJ189304-Colombia-2005, JN054256-SriLanka-2009, KF955446-Vietnam-2008, JX669473-Brazil-2001, JQ915076-French Polynesia:Tahiti-2009, FJ850114-Nicaragua-2005, FJ810415-Venezuela-2005, JN697058-Malaysia-2005, GQ398255-Singapore-2008 and AB074761-Japan-2001.

Although some recombination events might have occurred between some DENV-1 isolates (such as AB608788-Taiwan-1994 and AY726555-Myanmar-1998) as shown in Figure 2, recombination seems to be limited in these strains as only 9 out of 39 strains (23%) showed high probability to have recombination events (Table 3). Importantly, DENV-1-Jeddah-1-2011 isolate did not show any recombination event with any of the tested strains.

**Table 3:** **Recombination events positions**

**S.N.**	**Strain**	**Break point position**		**Av. P-Val with**
		**Beginning**	**Ending**	**RDP methods**
1	AB608788-Taiwan-1994	10544	1	9.268 × 10^-34^
2	JF459993-Myanmar-2002	99	2605	2.655 × 10^-66^
3	KJ189369-Mexico-2011	10456	15	8.658 × 10^-05^
4	KJ189368-Mexico-2012	10444	11	8.658 × 10^-05^
5	KF921948-Vietnam-2008	10284	1	1.182 × 10^-99^
6	KF184975-Angola-2013	10598	1667	1.705 × 10^-38^
7	AY726555-Myanmar-1998	8023	8300	3.853 × 10^-06^
8	JN903578-India-2007	3355	4395	3.881 × 10^-27^
9	JN903579-India-2008	5014	5658	1.716 × 10^-03^

**Figure 3:**
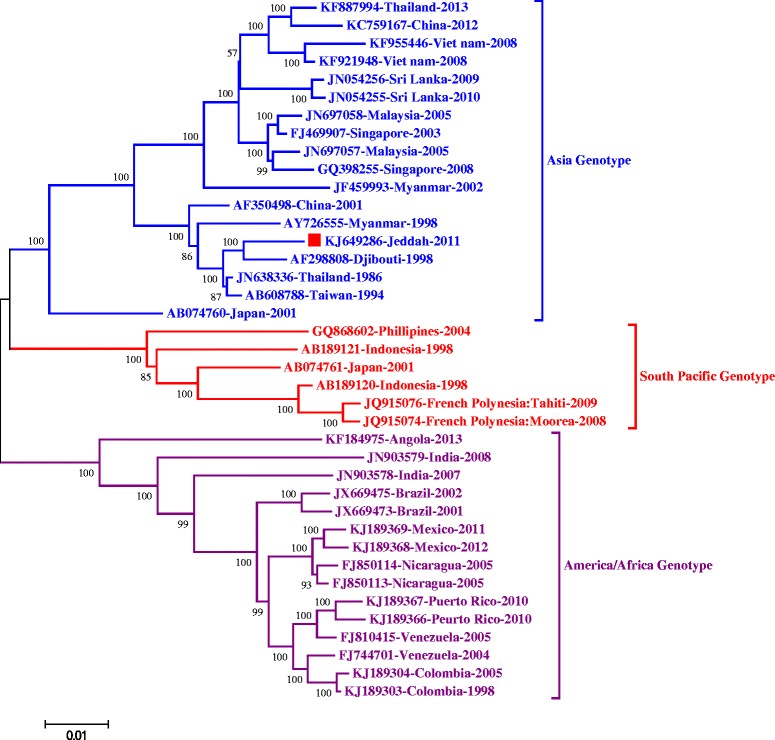
**Phylogenetic analysis of the full genome.** The phylogenetic tree was constructed for the full genome, as indicated in the Materials and methods. Tree was generated from nucleotide alignments of sequences from the culture isolate obtained from the patient in this study and highly similar DENV-1 sequences available in the GenBank database.

To gain further understanding of genetic relationship between DENV-1-Jeddah-1-2011 isolate and other isolates, phylogenetic analysis of the whole viral genome was performed (Figure 3). As expected, DENV-1-Jeddah-1-2011 isolate clustered with AF298808-Djibouti-1998, JN638336-Thailand-1986 and AB608788-Taiwan-1994 strains in addition to AY726555-Myanmar-1998 and AF350498-China-2001 strains.

### Envelope protein gene

**Figure 4:**
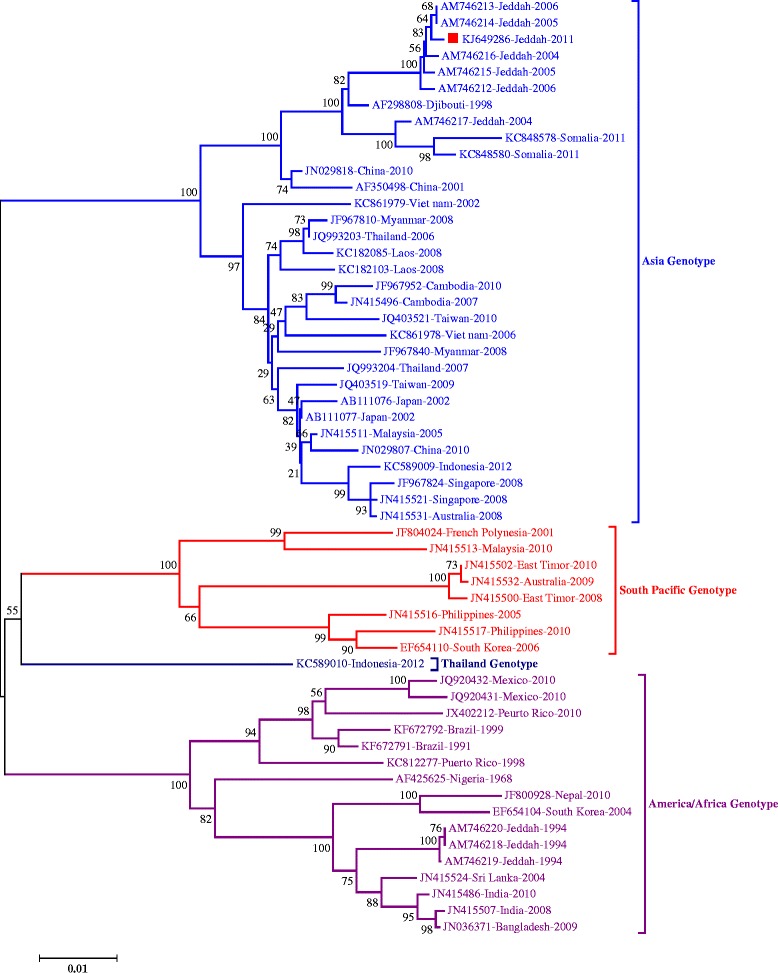
**Phylogenetic analysis of the full E gene.** The phylogenetic tree was constructed for the full E gene from DENV-1-Jeddah-1-2011 isolate and E genes of other isolates from diverse regions in the world.

Since DENV-1-Jeddah-1-2011 isolate genome represents the first reported complete genome of DENV-1 viruses from Saudi Arabia and that most available sequences in the GenBank are partial or complete coding sequences for the E protein only, we compared the E gene from DENV-1-Jeddah-1-2011 isolate to 57 selected DENV-1 isolates from various locations including isolates from Saudi Arabia and many other Islamic countries as shown in Table 4. Comparison of nt sand aa sequences of DENV-1-Jeddah-1-2011 isolate showed high similarity to previously reported isolates from Jeddah collected between 2004–2006 (AM746212-Jeddah-2006, AM746213-Jeddah-2006, AM746214-Jeddah-2005, AM746215-Jeddah-2005, AM746216-Jeddah-2004 and AM746217-Jeddah-2004). Interestingly, the E gene from two DENV-1 isolates from Somalia (KC848580-Somalia-2011 and KC848578-Somalia-2011) in addition to AF298808-Djibouti-1998 showed high similarity to DENV-1-Jeddah-1-2011 isolate. The lowest similarity (69.9% nt and 76.5% aa) was found with DENV-1 isolate (JN036391-Bangladesh-2006) (Table 4). As expected, phylogenetic analysis of the complete E gene of DENV-1-Jeddah-1-2011 isolate with geographically diverse DENV-1 isolates and other DENV-1 Jeddah isolates showed DENV-1-Jeddah-1-2011 clustering with some of the DENV-1 strains previously identified and reported from Jeddah together with the African AF298808-Djibouti-1998 strain and very closely related to KC848580-Somalia-2011, KC848578-Somalia-2011, AF350498-China-2011 and JN029818-China-2010 (Figure 4).

**Table 4:** **Nucleotide (NT) and amino acid (AA) identity of DENV-1-Jeddah-1-2011 E gene compared to closely related isolates**

**Strain**	**NT identity (%)**	**AA identity (%)**
AM746214-Jeddah-SA-2005	99.7	99.8
AM746213-Jeddah-SA-2006	99.7	99.8
AM746216-Jeddah-SA-2004	99.5	99.6
AM746215-Jeddah-SA-2005	99.5	99.6
AM746212-Jeddah-SA-2006	99.4	99.6
AF298808-Djibouti-1998	98.7	99.5
AM746217-Jeddah-SA-2004	98.0	99.1
JN029818-China-2010	97.7	99.3
KC848580-Somalia-2011	97.5	99.1
AF350498-China-2001	97.1	98.5
KC848578-Somalia-2011	97.1	98.9
JN415511-Malaysia-2005	95.6	98.1
AB111077-Japan-2002	95.5	98.1
JN415496-Cambodia-2007	95.4	97.9
JN029807-China-2010	95.3	98.3
JQ993203-Thailand-2006	95.3	97.7
AB111076-Japan-2002	95.3	97.5
JQ403519-Taiwan-2009	95.2	97.9
KC861979-Vietnam-2002	95.2	98.1
JQ993204-Thailand-2007	95.2	98.1
JF967810-Myanmar-2008	95.1	97.5
JF967952-Cambodia-2010	95.0	97.9
JF967840-Myanmar-2008	94.9	97.7
JN415531-Australia-2008	94.8	97.7
JN415521-Singapore-2008	94.8	97.5
KC861978-Vietnam-2006	94.7	97.3
JQ403521-Taiwan-2010	94.7	98.1
KC589009-Indonesia-2012	94.6	97.9
JF967824-Singapore-2008	94.6	97.5
KC589010-Indonesia-2012	91.7	96.7
KF672791-Brazil-1991	91.5	97.1
KC812277-Peurto Rico-1998	91.3	96.9
AF425625-Nigeria-1968	91.3	96.3
KF672792-Brazil-1999	91.2	97.1
KC182085-Laos-2008	91.1	92.5
JN415486-India-2010	91.1	96.9
AM746220-Jeddah-SA-1994	91.1	96.7
AM746219-Jeddah-SA-1994	91.1	96.7
AM746218-Jeddah-SA-1994	91.1	96.7
EF654110-South Korea-2006	91.0	97.3
JN036371-Bangladesh-2009	91.0	96.7
JN415524-Sri Lanka-2004	90.9	96.9
JN415516-Philippines2005	90.9	96.7
JN415507-India-2008	90.9	96.5
JX402212-Peurto Rico-2010	90.9	97.1
JN415517-Philippines-2010	90.8	96.9
JQ920432-Mexico-2010	90.7	96.7
JN415513-Malaysia-2010	90.5	96.9
JN415500-East Timor-2008	90.5	97.1
JQ920431-Mexico-2010	90.5	96.7
JF804024-French Polynesia-2001	90.5	92.5
JN415532-Australia-2009	90.4	97.1
JN415502-East Timor-2010	90.4	97.1
JF800928-Nepal-2010	90.3	96.1
EF654104-South Korea-2004	90.3	96.1
KC182103-Laos-2008	86.4	88.0
JN036391-Bangladesh-2006	69.9	76.5

## Discussion

DENV-1 has been circulating in several cities in the western and southern regions of Saudi Arabia since 1994 [13,14,18-20]. Phylogenetic analysis of partial genome sequences from several DENV-1 isolates from Jeddah showed two distinct genotypes [20]. Early in 1994, DENV-1 isolates from Jeddah were from the America/Africa genotype. However, in 2004, circulating DENV-1 viruses were found to be from the Asian genotype in which they clustered with the African AF298808-Djibouti-1998 strain [20]. The later genotype continued to circulate in Saudi Arabia until 2006 [20]. In this study, we were interested to extend previous studies and to investigate the genetic relationship between circulating DENV-1 viruses in Saudi Arabia and other parts of the world, with emphasis on Islamic countries due to the religious ties between these countries and the holy places in Makkah and Al-Madinah during Hajj pilgrimage and Umrah seasons. Jeddah, being the hub of transportation of Muslims coming in and out of the holy places in Makkah and Al-Madinah, is of particular interest since the large influx of pilgrims going through Jeddah could be a major source of virus transportation to the western region of the country.

Sequence alignment and phylogenetic analysis of the full-length genome of DENV-1-Jeddah-1-2011 isolate showed a very close relationship between this isolate and the African AF298808-Djibouti-1998 strain in the Asian genotype, suggesting that this genotype was circulating at least until 2011 in Saudi Arabia since its introduction. The observed genetic variation in DENV-1-Jeddah-1-2011 isolate compared to other related strains, both at the nt and aa levels, as well as the deletion in the 3’-UTR are most likely due to local evolution and adaptation of this virus. Analysis of the deduced aa sequence of the DENV-1-Jeddah-1-2011 isolate compared to the most closely related strain (AF298808-Djibouti-1998), showed that most of the aa substitution were in the NS5 protein (12/26) with only two changes in the E protein (Table 2), suggesting that using the E gene only for phylogenetic analysis might not be the best approach to study genetic relationship between DENVs.

Nonetheless, since most of the reported DENV sequences in the Genbank, including those from Saudi Arabia, are from the E region, comparing the E gene from DENV-1-Jeddah-1-2011 to these sequences should give more insight into the genetic relatedness of these strains. Analysis of the E gene of DENV-1-Jeddah-1-2011 isolate compared to other reported sequences showed high similarity and close clustering of this strain with most recent isolates collected from Jeddah between 2004 and 2006 in addition to the African AF298808-Djibouti-1998 strain (Figure 4). Interestingly, these viruses were closely related to two recent isolates from Somalia (KC848580-Somalia-2011 and KC848578-Somalia-2011) and China (AF350498-China-2011 and JN029818-China-2010) (Table 4, and Figure 4). The high similarity between DENV-1-Jeddah-1-2011, the African AF298808-Djibouti-1998 and the 2004-2006 isolates from Jeddah suggest that DENV-1 strains from the Asian genotype might have been imported with African pilgrims long before 2004. Additionally, the similarity of these strains to recent isolates from Somalia suggests the widespread of this genotype in this region most probably due to importation with Hajj pilgrims. Interestingly, the African AF298808-Djibouti-1998 strain is more genetically related to Asian isolates than to African isolates [21] indicating that DENV strains can be transmitted and sustained in countries far from their geographic origin.

Detection of two DENV-1 genotypes in Saudi Arabia in the past two decades as well as the several outbreaks reported in Makkah, Al-Madinah and Jeddah [15,18-20] suggest that pilgrims could play a major role in the introduction and distribution of DENV serotypes in Saudi Arabia especially in the two holy cities of Makkah and Al-Madinah as well as Jeddah. While most recent DENV-1 isolates from Jeddah belong to the Asian genotype, it is not known if this genotype completely replaced previously circulating America/Africa genotype or both genotypes are co-circulating due to the very limited number of published studies on DENVs from Saudi Arabia.

Introduction of new DENV viruses can lead to the emergence of recombinant strains which might have a great impact on the epidemiological and clinical outcomes. Interestingly, Tolou and colleagues reported that DENV-1 Singapore S275/90 strain is a product of a recombination event between viruses from two distinct lineages in which one lineage includes an African strain isolated in Abidjan in Ivory Coast and the other includes isolates from Djibouti (AF298808-Djibouti-1998) and the closely related isolate from Cambodia [21]. Identification of such events depends on the availability of full genome sequences of DENV strains from various regions which is missing in Saudi Arabia. Phylogenetic analysis of DENV-1-Jeddah-1-2011 isolate sequence, Asian genotype, did not show any genetic relations with the other DENV-1 genotypes including the America/Africa genotype which was circulating in Jeddah area since 1994. Furthermore, recombination analysis showed no such events in the DENV-1-Jeddah-1-2011 isolate.

## Conclusion

In this study, we report the first complete genome sequence of DENV-1 isolate from Saudi Arabia which can represent a reference genome for future genetic studies of DENV-1 viruses. Sequence alignment and phylogenetic analysis of this isolate suggest that the Asian genotype might have been introduced into Saudi Arabia long before 2004 from the African continent probably through pilgrims. Pilgrims from all over the world, particularly dengue endemic regions, travel through Jeddah to Makkah and Al-Madinah in Saudi Arabia to perform Hajj and Umrah which can lead to the introduction and spread of new strains of DENV into Saudi Arabia as well as other geographical regions. While the high influx of pilgrims coming from DENV endemic areas to Jeddah and the holy places warrants further studies of more samples to have a clear picture about other circulating strains, availability of this complete genome sequence would serve as a reference for such future epidemiological, virological and genetic studies of DENV-1 viruses. Nonetheless, measures to screen pilgrims from DENV endemic areas might be critical to reduce the spread of the disease and the economic and healthcare burdens of the disease in Saudi Arabia and other parts of the world.

## Materials and methods

### Samples

Plasma and serum samples were collected from a female patient with suspected DENV infection at King Abdulaziz University Hospital and stored at −80°C until testing. Ethical approval was obtained from the Biomedical Research Ethical Committee at at King Abdulaziz University Hospital, King Abdulaziz University (reference # 19-14).

### ELISA

Serum sample was screened for anti-DENV IgM and IgG antibodies using Panbio® Dengue IgM and IgG Capture ELISA Kits (Panbio, Australia) according to the manufacturer’s instructions.

### RNA extraction and RT-PCR

Viral RNA was extracted from plasma or culture supernatant using QIAamp viral RNA mini kit (Qiagen, Germany) according to manufacturer’s instructions. Eluted RNA was screened for DENV-1 RNA by real time RT-PCR using primers and probes previously described [22].

### Virus isolation by cell culture

Mosquito *Aedes albopictus* C6/36 cells maintained in complete Dulbecco's Modified Eagle's Medium (DMEM) supplemented with 2% fetal bovine serum (FCS), 2 mM L-glutamine, 1.5 g/l sodium bicarbonate, 1.0 mM sodium pyruvate, 100 U/ml penicillin and 100 μg/ml streptomycin. Cells were incubated in a humidified atmosphere at 28°C in 5% CO_2_. Cells were inoculated with 100 μL of patient plasma and examined daily for CPE. After CPE is observed, cell culture supernatants were collected and analyzed by real time RT-PCR. DENV-1 RNA-positive culture supernatants were used for whole viral genome sequencing.

### DENV genome sequencing

Viral RNA extracted from culture supernatant was subjected to RT-PCR amplification using primer pairs covering the whole length of the viral genome using an ABI Veriti thermal cycler (Applied Biosystems, USA) as previously described [23]. The RT-PCR amplified fragments (600-800 bp), which cover the whole genome sequence and overlap in common regions, were excised from agarose gel and purified using QIAquick Gel Extraction Kit (Qiagen, Germany) according to manufacturer’s instructions. The products were subjected to cycle sequencing on an ABI 3500 Automatic Sequencer (Applied Biosystems, USA) using the Bigdye Terminator V3.1 Reaction Cycle Kit (ABI, Germany) according to manufacturer’s instructions. The complete viral sequence was assembled and deposited in the GenBank with the following accession number (GenBank: KJ649286; DENV-1-Jeddah-1-2011).

### Sequence alignment, phylogenetic and recombination analysis

The complete genome of DENV-1-Jeddah-1-2011 isolate was initially searched for similarity using BLAST software (http://www.ncbi.nlm.nih.gov/BLAST/). DENV-1 sequences with high similarity were selected for further analysis. Specifically, sequences were multiply aligned using ClustalW and nt sequences identity matrix as well as aa substitutions were analyzed. Phylogenetic analysis and distance calculations were performed using the MEGA v.5 software with the Neighbor-Joining method of the Maximum Composite Likelihood model, gamma-distributed rates among sites with 1,000 bootstrap replicates. Recombination analysis was conducted with RDP3 package with P-value < 0.05 [24].
